# Editorial: Nanoparticles in Cancer Therapy-Novel Concepts, Mechanisms, and Applications

**DOI:** 10.3389/fphar.2018.01552

**Published:** 2019-01-22

**Authors:** Qingxin Mu, Bing Yan

**Affiliations:** ^1^Department of Pharmaceutics, University of Washington, Seattle, WA, United States; ^2^Key Laboratory for Water Quality and Conservation of the Pearl River Delta, Ministry of Education, Institute of Environmental Research at Greater Bay, Guangzhou University, Guangzhou, China; ^3^School of Environmental Science and Engineering, Shandong University, Jinan, China

**Keywords:** nanoparticles, nanomedicine, nanotechnology, drug delivery, cancer therapy

Since the invention of nanomedicine decades ago (Drexler et al., 1991; Weber, 1999; Kalb, 2000; Voss, 2000), considerable progresses have been made, especially with cancer as a target (Jain and Stylianopoulos, 2010; Shi et al., 2017). Active research spread from fundamental research to clinical investigations. The topic “Nanoparticles in Cancer Therapy-Novel Concepts, Mechanisms and Applications” intends to cover several important aspects in this field including nanocarrier development, gene delivery, intrinsically active nanoparticles (NPs), tumor microenvironment, immunology, and toxicity.

The mostly applied biocompatible nanocarriers in clinical trials are protein, lipid, and polymer-based materials. In this aspect, three reviews are included. Gou et al. comprehensively summarized protein-based nanoformulations for cancer theranostics. They discussed nanocarriers composed of albumin, ferritin, gelatin, and transferrin, which are the major materials investigated. Imaging modalities in addition to these nanocarriers are also discussed including near-IR fluorescence, magnetic resonance imaging, positron emission tomography, computed tomography, and photoacoustic imaging. The authors also analyzed challenges these formulations face such as reproducibility, colloidal stability in biological environments, drug-loading efficiency, and reticuloendothelial system accumulation. Rezvantalab et al. systematically reviewed the PLGA-based NPs in cancer treatment. The review article gives an overview of properties and preparation methods of PLGA NPs. Emphasis is also given to the passive and active tumor targeting, and combination therapy. The authors argued that researchers need to critically reflect on the clinical feasibility and give consideration to not only the optimization of size, drug loading and drug release, but also biocompatibility, pharmaceutical upscaling and batch-to-batch reproducibility. The authors also encourage more pharmacokinetic and pharmacodynamic understanding of NPs, and applicability of external stimuli for combination therapy. Raut et al. provides an in-depth review of recombinant high-density lipoprotein (rHDL)-based drug delivery systems in cancer therapy. The rHDL NPs are inherently capable of overcoming several biological barriers to cancer therapy. The small size, intrinsic targeting ability, endosomal escape, and safety in animals and humans make this platform highly attractive for chemotherapy drugs which suffer from off-target toxicity issues. In their review, detailed discussion was given in the following aspects: interactions of rHDL NPs with blood and immune cells, hemorheology, and blood vessel fluid dynamics, extravasation, cellular membrane transport, and endosomal escape, and drug resistance.

Polymeric micelles are a popular type of nanocarriers for drug delivery due to their biocompatibility, multifunctionality, and controllable release. In this topic, Qi et al. synthesized triblock copolymers PEG-DiHyd-PLA containing hydrazone bond. These copolymers can self-assemble into micelles with uniformed size below 100 nm and narrow size distribution. The size underwent obvious changes in mildly acidic environments while kept unchanged in neutral condition. Model drug doxorubicin was successfully loaded into the micelles and presented a rapid and complete drug release in acidic condition (pH 5.0). The drug loaded NPs possessed high anti-tumor activity to kill the cancer cells but minimum toxicity to normal cells. Li et al. synthesized an ibuprofen-based amphiphilic diblock copolymer (POEG-b-PVBIBU) via reversible addition fragmentation transfer polymerization of the drug-based vinyl monomer. The diblock copolymer was able to self-assemble into prodrug nanomicelles and load anti-cancer drugs with high capacity such as doxorubicin, paclitaxel and docetaxel. The drug containing polymeric micelles were more effective in inhibiting the tumor growth than free DOX *in vivo*.

Li et al. achieved RNA interference for cancer therapy using TDAPEI, a synthetic derivative of low-molecular-weight polyethylenimine (PEI) as a carrier of plasmid DNA encoding VEGF shRNA. The polymers were cross-linked with imine bonds by the conjugation of branched PEI (1.8 kDa) and 2,5-thiophenedicarboxaldehyde (TDA). The nanocomplex showed effective *in vivo* anti-tumor efficacy and gene silencing comparable to PEI (25 kDa) as carrier but greater biocompatibility.

NPs can also be intrinsically active for cancer therapy. Corsi et al. reviewed the biological effects of cerium oxide NPs. Despite cerium oxide NPs (CNPs) were found to exert strong anticancer activities which is a characteristic generally attributed to CNPs redox activity, other studies however reported non-redox mechanisms. The authors recently demonstrated that the radio-sensitizing effect of CNPs on human keratinocytes is independent from the redox switch. Mechanisms involving particle dissolution with release of toxic Ce^4+^ atoms, or differential inhibition of the catalase vs. superoxide dismutase-mimetic activity with accumulation of H_2_O_2_ have been proposed, explaining such intriguing findings only partially.

Another example of intrinsically active NPs are through interacting with human immune systems. Jia et al. discussed the interactions of NPs with dendritic cells and its applications in cancer therapy. Targeting dendritic cells (DCs) by nanotechnology stands as a promising strategy for cancer immunotherapy. The physicochemical properties of NPs influence their interactions with DCs, thus altering the immune outcome of DCs by changing their functions in the processes of maturation, homing, antigen processing, and antigen presentation. In their review, Jia et al. summarized the recent progress in targeting DCs using NPs as a drug delivery carrier in cancer immunotherapy, the recognition of NPs by DCs, and the ways the physicochemical properties of NPs affect DCs' functions. The molecular pathways in DCs that are affected by NPs were also discussed.

The tumor microenvironment plays a vital role in regulating nano-chemotherapeutics distribution and their biological effects. Fernandes et al. summarized the barriers in tumor microenvironment, their consequential effects on chemotherapeutics, considerations to improve nano-chemotherapeutics delivery and combinatorial strategies to overcome acquired resistance induced by tumor microenvironment. Nanotechnology based approach as well as combinatorial approaches based on ligand-mediated, redox-responsive, and enzyme-mediated strategies have been discussed in their review.

Nanotechnology has also shown several advantages over widely used traditional methods in uro-oncology. For example, different types of NPs improve the solubility of poorly soluble drugs, and multifunctional NPs have good specificity toward prostate, renal, and bladder cancer. Moreover, nanotechnology can also combine with other novel technologies to further enhance efficacy. As our understanding of nanotechnologies grows, additional opportunities to improve the diagnosis and treatment of urological cancer are expected to arise. In their review, He et al. focused on nanotechnologies with potential applications in urological cancer therapy and highlighted clinical areas that would benefit from nanomedicine therapy.

As concerns about the toxicity of nanomaterials are growing, a safety evaluation become a routine assessment nowadays as a prerequisite for nanomedicine. Chen et al. showed that carboxyl and amine-modified InP/ZnS quantum dots were able to enter the cells with high uptake efficiency. High doses of NPs caused the cell viability to decrease, and carboxyl or amine-modified quantum dots appeared to be more toxic than hydroxyl modified ones. In addition, all these NPs were able to induce intracellular reactive oxygen species generation and apoptosis. These results suggested that proper caution should be taken for the safe application of nanomedicine.

Although this topic included some key advances, we recognize that this field is growing very rapidly and our collection has not covered all aspects. In recent years, nanotechnology has shown many advantages over conventional approaches for cancer diagnosis, treatment and prevention, and clinical trials are being implemented every year (Tran et al., 2017). However, challenges are still overwhelming especially in human trials, formulation, and regulatory issues. With new advancements in materials chemistry, nanoscience, biology and medicine, one can envision that devastating cancer will be curable some day and in that achievement, the nanoscale approaches will have played an important role.

## Author Contributions

All authors listed have made a substantial, direct and intellectual contribution to the work, and approved it for publication.

### Conflict of Interest Statement

The authors declare that the research was conducted in the absence of any commercial or financial relationships that could be construed as a potential conflict of interest.
